# Methodology and challenges for harmonization of nutritional data from seven historical studies

**DOI:** 10.1186/s12937-024-00976-8

**Published:** 2024-08-06

**Authors:** Sivan Ben Avraham, Angela Chetrit, Nirit Agay, Laurence S. Freedman, Walid Saliba, Uri Goldbourt, Lital Keinan-Boker, Ofra Kalter-Leibovici, Danit R. Shahar, Lizie Kimron, Rachel Dankner

**Affiliations:** 1 Gertner Institute for Epidemiology and Health Policy Research, Sheba Medical Center, Ramat Gan, Israel; 2 Department of Community Medicine and Epidemiology, Lady Davis Carmel Medical Center, Haifa, Israel; 3 Ruth and Bruce Rappaport Faculty of Medicine, Technion-Israel Institute of Technology, Haifa, Israel; 4 Department for Epidemiology and Preventive Medicine, School of Public Health, Sackler Faculty of Medicine, Tel Aviv University, Tel Aviv, Israel; 5 Israel Center for Disease Control, Israel Ministry of Health, Ramat Gan, Israel; 6 School of Public Health, University of Haifa, Haifa, Israel; 7 Department of Epidemiology, Biostatistics and Community Health Sciences, The International Center of Health Innovation & Nutrition, Ben-Gurion University of the Negev, Be’er Sheva, Israel

**Keywords:** Historical cohort, Nutritional data, Methodology, Harmonization

## Abstract

**Background:**

Collection of detailed dietary data is labor intensive and expensive, harmonization of existing data sets has been proposed as an effective tool for research questions in which individual studies are underpowered**.**

**Methods:**

In this paper, we describe the methodology used to retrospectively harmonize nutritional data from multiple sources, based on the individual participant data of all available studies, which collected nutritional data in Israel between 1963 and 2014. This collaboration was established in order to study the association of red and processed meat with colorectal cancer. Two types of nutritional questionnaires, the Food Frequency Questionnaires (FFQ) and the 24-h dietary recall (24HR recall), and different food composition tables, were used by the participating studies. The main exposure of interest included type of meat (total meat, red meat, and poultry) and level of processing.

**Results:**

A total of 29,560 Israeli men and women were enrolled. In studies using FFQ,the weighted mean intakes of total, red, processed meat, and poultry were 95, 27, 37 and 58 gr/day and 92, 25, 10, and 66 gr/day in studies using 24HR recall, respectively.. Despite several methodological challenges, we successfully harmonized nutritional data from the different studies.

**Conclusions:**

This paper emphasizes the significance and feasibility of harmonization of previously collected nutritional data, offering an opportunity to examine associations between a range of dietary exposures and the outcome of interest, while minimizing costs and time in epidemiological studies.

**Supplementary Information:**

The online version contains supplementary material available at 10.1186/s12937-024-00976-8.

## Introduction

The pooling and harmonization of existing data sets has been proposed as an effective and important tool for research questions in which individual studies are underpowered and for the study of rare outcomes [1].

The dietary exposure is complex and in recent years nutritional studies shifted their focus from single nutrients to foods, dietary patterns and attributes of the diet such as level of processing [2, 3]. Unlike nutrients, that are a universal element of the diet, the types of foods people eat and food patterns vary greatly, and require large sample sizes and diverse populations to represent this heterogeneity [4, 5]. A recent review highlighted the importance of harmonization in nutritional studies, as many studies use relatively small populations with limited power and generalizability [6].

These methodological challenges are even more pronounced with research questions dealing with the etiology of chronic diseases with extended latency periods, requiring long follow-up [7].

We established a collaboration, calling out to all potential partners with historical nutritional data bases collected in the country over the past decades in order to study the association between nutrition and colorectal cancer.

The aim of this paper is to describe the methodology used to harmonize nutritional data from multiple sources with diverse nutritional questionnaires and nutrient databases. A secondary aim is to describe characteristics of the participating studies and dietary intake of participants.

## Methods

This is a collaborative historical cohort study, based on the individual participant data of 7 studies (*N* = 29,560), which collected nutritional data between 1963 and 2014.

To be included, studies had to be conducted in Israel, collected a single detailed dietary intake data, and have identified records to enable linkage with the Israeli Cancer Registry (INCR) and the central population registry system for vital status using the unique identifying number each Israeli citizen holds. Principal investigators were contacted to confirm the eligibility of their studies and their willingness to share their data. After obtaining an Institutional Review Board (IRB) approval, each principal investigator transferred a study file to the study center with a set of variables based on a common dataset identified by the study team. Participating studies were originally of cohort, cross-sectional or case–control design; for case–control studies, we only included data of the control subjects free of cancer, the outcome of interest, by the time of the nutritional interview.

In addition to dietary data, all studies collected information on sociodemographic, lifestyle and health characteristics of the participants. Harmonization of these variables was performed as well, yielding to comparable datasets.

The following studies were included in the collaboration (see Table 1 for additional details):

1. The Israeli Cohort Study of Glucose Intolerance, Obesity and Hypertension (GOH) [8, 9];
2. The Israeli Ischemic Heart Disease study (IIHD) [10, 11];
3. The Hadera District Arabs and Jews Study (HDS) [12];
4. The Mabat Zahav survey [13, 14];
5. The ovarian cancer study (Ovary) [15];
6. Northern Israel Cancer Case Control studies (NICCCS) [16, 17];
7. The Negev Nutritional Study (NNS) [18, 19].

**Table 1:** Description of the Participating Study' Characteristics and their Nutritional Questionnaires

Characteristic	IIHD	GOH	Ovary	NNS	NICCCS	HDS	Mabat Zahav
Reference	10, 11	8, 9	15	18, 19	16, 17	12	13, 14
Design	Cohort	Cohort	Case–Control	Cohort	Case–Control	Cohort	Cohort^a^
Sex	Men	Men and women	Women	Men and women	Men and women	Men and women	Men and women,
Inclusion criteria	Jewish men, 40 years and above, Government or municipal workers of the 3 largest cities (Haifa, Tel Aviv and Jerusalem)	Randomly sampled from the Central Population Registry, according to birth year	Adult women with no history of cancer	Random sample of the Jewish adult (35 years and above) South district (Negev) population	Healthy controls of a population-based, matched case–control study	A random, population-based sample stratified equally across gender, ethnicity, and 10-y age-groups	National Health and Nutrition Survey designed to represent the Israeli Jewish and Arab elderly population of 65 years and older
Study Population	A stratified sample of the six main areas of birth (Eastern Europe, Central Europe, South Eastern Europe, Israel, the Middle East and North Africa	A stratified sample of the four main ethnic origins, Men and Women, Jewish only	Controls of a nationwide case–control study on ovarian cancer, Jewish only	Population from the Negev area including immigrants from the former USSR; Men and women Jewish only	Population from Israel's northern area. Controls of colon cancer cases; Jewish and Arab participants; Men and women	Hadera district pop., designed to include equal numbers of Arab and Jewish participants men and women	A representative sample of 65 + y old Israeli population Men and women
Nutritional assessment time	1963–1967	1982	1994–1996	1998–2000	1998–2014	2002–2007	2005–2006
Participants with available dietary data (n]	10,059 (1058 with full food item data)	632	1763	986	11,658	1098	1751
Age range at recruitment (years]	40–65	41–70	21–85	35 +	21 +	25–74	65 +
Nutritional assessment type	Sq-FFQ	q-FFQ	q-FFQ	24HR recall	Sq-FFQ	q-FFQ	24HR recall
Number of food and beverage items in FFQ/n of foods reported in 24HR recall	214	240	180	2435	187	240	2250
Food composition table	Based on local manufacturers data, composition tables of locally grown food and international composition tables	Based on the British McCance and Widdowson's tables, local manufacturers data and other sources	Based on the British McCance and Widdowson's tables, local manufactures data and other sources	Based on the USDA database, local manufacturers data and other sources	Based on the Israeli Ministry of health food composition tables, local food manufacturers, and international composition tables	Based on the British McCance and Widdowson's tables, local manufacturers data and supplemented with common Arabic foods and dishes	Based on The Israeli Ministry of health food composition tables

*Abbreviations*: *IIHD* Israel Ischemic Heart Study, *GOH* Glucose Intolerance, Obesity, and Hypertension study, *NNS* the Negev Nutritional Study, *NICCCS* Northern Israel Cancer Case Control Studies, *HDS* Hadera District Study, *sq-FFQ* Semi-quantitative food frequency questionnaire. *q-FFQ* quantitative food frequency questionnaire

^a^Originally designed as a survey

Twelve individuals were found to take part in more than one of the participating studies. Their information from the study with more complete data was included in the current collaborative analysis.

### Study data

A unified coding system for non-dietary variables, which may serve as potential confounding factors, was developed mapping the available variables among studies and data coding was standardized accordingly. These variables included: date of nutritional interview, age, sex, ethnicity, country of birth, education, marital status, cigarette smoking habits, body weight, height, or calculated Body Mass Index (BMI), and physical activity. Information on lifestyle (physical activity and smoking) was available for 6 of the 7 studies**.**

#### Nutritional data harmonization

There were several differences regarding nutritional data assessment between the studies included in the current analysis: type of nutritional questionnaire, the foods and nutrient databases, and periods of data collection.

#### Nutritional questionnaires and food composition databases

The studies included in this collaboration used three types of nutritional questionnaires and five nutritional databases for calculation of nutrient intake. The types of questionnaires used were a semi-quantitative Food Frequency Questionnaires (sq-FFQ), a quantitative Food Frequency Questionnaire (q-FFQ) and a 24HR recall.

#### Food and nutrient composition databases

Differences in food composition databases included variations in nutrient composition and in portion sizes defined. Some of these differences reflect characteristics of the food composition and food supply in the period in which the original study was conducted. In order to represent these characteristics accurately, nutrient composition was calculated (or received pre-calculated from the PI) for each study using its original database.

Harmonization of the data was performed on the food and food group level for the specific purpose of studying meat intake. A nutritional epidemiologist reviewed food level dietary data using the original data dictionaries and descriptive statistics, portion sizes were translated into grams and a common categorization system for foods was created. The system was used to group single foods into 22 common food groups with an emphasis on food groups of interest to the project (red meat, processed meat and poultry). Meat was sub-categorized by level of processing (unprocessed, processed and ultra-processed) (see Table 4), Organ meats represented a separate group. Red meat included beef, veal, pork, lamb, mutton, and goat, in accordance with the IARC definition [1]. Processed meat was divided into two sub-groups within each meat type: processed, which included items such as hamburger and breaded and fried chicken breast, and ultra-processed, which included items such as sausages, hotdogs, pastrami and chicken nuggets, in line with the NOVA classification [7]. In addition, to avoid overestimation of meat intake [20], composite dishes that include meat (i.e. meat-stuffed vegetables etc.) were separated into sub-groups by meat type. The meat content was then calculated according to its relative share of the dish (usually 30% of weight). The meat content of composite meat dishes was included in the unprocessed meat sub group.

First, reported food consumption was converted into average daily amounts consumed based on frequencies, number of portions and portion sizes. For the FFQ, seasonal items were adapted to the length of the Israeli season in which the item is mostly available. Energy intake and several macro and micronutrients intakes were calculated for each food item using information on food composition from international and local sources, multiplying the quantity consumed of each food item by the values of energy intake and micro and macronutrients in 100 g of that food item. Secondly, the foods were grouped to 8 subgroups of meat and fish items and 14 groups of other food items (fruits, vegetables, bread and cereals, milk and milk products, eggs, legumes, nuts and seeds, sweets, sugar sweetened beverages, artificially sweetened sweets and beverages, alcoholic beverages, ethnic dishes, spreads, sauces and spices). Working files were built for each study including for each food group selected macro and micronutrients intakes and their densities, namely: energy intake, carbohydrates, protein, total and saturated fat, fibers, cholesterol, alcohol, calcium iron and folic acid (22 food groups times (10 nutrients + 10 densities + energy intake)). Building the database in this way allowed exploring of the nutritional exposure both in terms of intake of a food group or by percent energy, as well as by its contribution of specific nutrients (i.e. iron from meat, fibers from fruit etc.). Descriptive statistics and frequency tables were used to check for errors, inaccuracies and missing data, which were then discussed with the PIs and updated when possible.

A complete dataset including individual level dietary, socio-demographic and lifestyle information was generated for each participating study. Computing of variables, building the working files and data analyses were performed using SAS version 9.4.

### Statistical analysis

The current study presents descriptive statistics of sociodemographic and anthropometric characteristics of each study, as well as nutritional data.

Meat consumption variables were studied by quartiles of total meat, red meat, processed meat and poultry, and median values of each quartile are presented according to study (Table 3). In addition, meat consumption, nutrient intake and dietary intake variables were studied as continuous variables. Each study provides a mean consumption with a different precision, where the precision depends partly on the sample size and partly on the variance of the dietary intakes as reported in that study. Therefore, in order to characterize meat consumption of the pooled study population, weighting method, ensuring that the estimates with higher precision receive higher weight was applied as follows: in each study, the mean and standard error for each meat consumption variable, a weighted mean was calculated according to the following formula $w=\frac{1}{{se}^{2}}$ (Table 4) and its standard error as 1 divided by the square root of the sum of weights ($\frac{1}{\sqrt{\sum w}}$). Weighted means were calculated for men and women separately, and by type of study questionnaire (FFQ or 24HR recall). A weighted mean was also calculated for each quartile of meat consumption, as described above. A comparison of selected nutrients intake by total meat quartiles was done via a test for linear trend across quartiles (Table 5).

## Results

Table 1. includes a summary of the different study participants' characteristics. Five of the studies included both men and women, one included men only and one women only. Both Jewish and Arab participants were included in three of the studies.

**Fig. 1:**
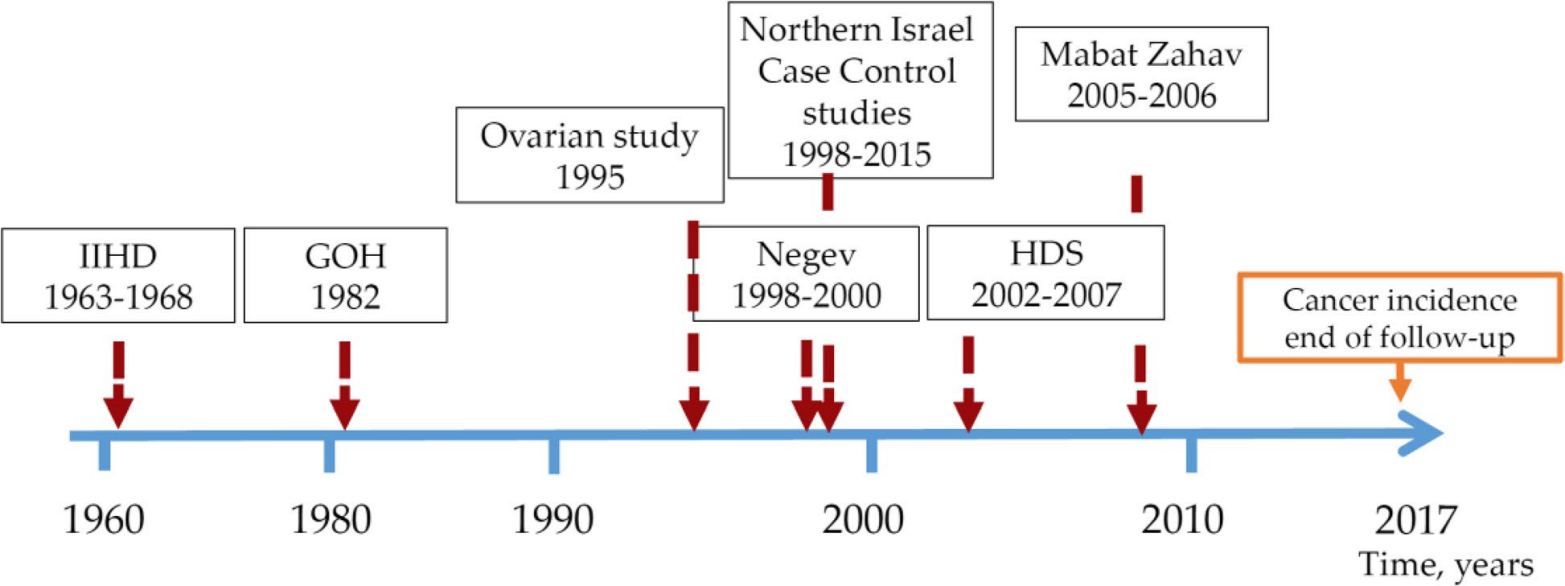
Schematic description of a historical collaborative nutritional study

Recruitment to the different studies spanned from 1963 until 2014 (Fig. 1), and the nutritional questionnaires were FFQ in five of the studies and 24HR recalls in two. Four different food composition tables were used for the nutrient analysis, three studies used a variation of The Mccance and Widdowson's Composition of Foods [21], two studies had costume built their databases based on several sources and two others were based on the Israeli ministry of health food composition tables [20]. These tables provided the relevant food composition available from the actual time period of the specific study.

Demographic characteristics of the population by study are displayed in Table 2. The mean age at study start ranged from 49.3 ± 6.3 years in IIHD to 74.6 ± 6.2 in Mabat Zahav. Rates of current smoking and of less than 12 years education were highest in IIHD. Rates of obesity (BMI ≥ 30 kg/M^2^) were lowest in the IIHD and GOH studies and highest in the HDS and Mabat Zahav.

**Table 2:** Baseline demographic characteristics of the study population by study

	**Study**						
Characteristic	**IIHD** *n* = 10,059	**GOH** *n* = 632	**Ovary** *n* = 1,763	**NNS** *n* = 986	**NICCCS** *n* = 13,357	**Mabat Zahav** *n* = 1,751	**HDS** *n* = 1,098
Sex, male, n (%)	10,059 (100)	305 (48.3)	0 (0)	451 (45.7)	3,072 (23)	819 (46.8)	549 (50)
Age, mean ± SD	49.3 ± 6.3	54.7 ± 8.1	57.5 ± 14.0	57.9 ± 12.8	65.1 ± 13.3	74.6 ± 6.2	50.9 ± 14.3
Smoking status, n (%)							
Never	3,158 (31.4)	379 (60)	1,193 (67.7)	-	8,242 (61.7)	945 (54)	608 (55.4)
Current	5,217 (51.9)	182 (28.8)	327 (18.5)	-	1,997 (15.0)	190 (10.9)	306 (27.9)
Past	1,677 (16.7)	62 (9.8)	239 (13.6)	-	3,097 (23.2)	607 (34.7)	184 (16.8)
Missing	7 (0.1)	9 (1.4)	4 (0.2)	-	21 (0.2)	9 (0.5)	
Physical activity, n (%)							
Yes	7,390 (73.5)	494 (78.2)	1,113 (63.1)	-	5,685 (42.6)	1,032 (40)	447 (40.7)
No	2,229 (22.2)	22 (3.5)	645 (36.6)	-	7,651 (57.3)	708 (59)	651 (59.3)
Missing	440 (4.4)	116 (18.4)	5 (0.3)	-	21 (0.2)	11 (1)	-
BMI, Kg/m^2^, n (%)							
< 20	449 (4.5)	12 (1.9)	116 (6.6)	42 (4.3)	514 (3.8)	20 (1.1)	37 (3.4)
20–24.9	3,752 (37.3)	259 (41.0)	627 (35.6)	331 (33.6)	4,126 (30.9)	257 (14.7)	228 (20.8)
25–29.9	4,888 (48.6)	256 (40.5)	523 (29.7)	380 (38.5)	4,874 (36.5)	697 (39.8)	435 (39.6)
≥ 30	945 (9.4)	96 (15.2)	301 (17.1)	205 (20.8)	3,295 (24.7)	601 (34.3)	379 (34.5)
Missing	25 (0.2)	9 (1.4)	196 (11.1)	28 (2.8)	548 (4.1)	176 (10.1)	19 (1.7)
Education, years, n (%)							
< 12	6,162 (61.4)	-	667 (37.8)	383 (38.8)	4,058 (30.4)	852 (48.7)	535 (48.7)
12	1,635 (16.3)	-	370 (21.0)	239 (24.2)	2,662 (19.9)	298 (17)	206 (18.8)
> 12	2,226 (22.1)	-	718 (40.7)	362 (36.7)	6,039 (45.2)	593 (33.9)	354 (32.2)
Missing	36 (0.4)	-	8 (0.5)	2 (0.2)	598 (4.5)	8 (0.5)	3 (0.3)
Ethnicity*, n (%)							
Europe/America	5,037 (50.1)	260 (41.1)	1,008 (57.2)	0	5,496 (41.1)	782 (44.7)	230 (20.9)
Africa/Asia	3,591 (35.7)	372 (58.9)	309 (17.5)	0	2,406 (18.0)	413 (23.6)	102 (9.2)
Israel	1,431 (14.2)	0	446 (25.3)	190 (19.3)	3,686 (26.8)	267 (15.2)	218 (19.8)
Israeli Arab /others	0	0	0	0	1,742 (13.0)	287 (16.4)	548 (49.9)
Missing	0	0	0	796 (80.7)	127 (1.0)	2 (0.1)	

^*^Europe/America, Africa/Asia and Israel refer to Jewish participants only.Table abbreviations, *IIHD* Israel Ischemic Heart Study, *GOH* Glucose Intolerance, Obesity, and Hypertension study; NNS the Negev Nutritional Study, *NICCCS* Northern Israel Cancer Case Control Studies, *HDS* Hadera District Study

Meat consumption was categorized according to quartiles. The median value of each quartile of total meat intake and intakes of poultry, red and processed meat (gr/day) by study and type of nutritional assessment, are presented in Table 3. In studies using FFQ, 97% to 100% of participants reported consuming any meat; intakes of total meat in the fourth (highest) quartile ranged from 117 gr/day in the Ovary study and went up to 246 gr/day in the GOH study. In studies using 24HR recalls, total meat intakes ranged from 0 to 191gr/day in Q1 (lowest) and Q4, respectively. Of the meat sub-categories, intakes of poultry were highest across all studies with median intakes in Q4 ranging from 90 to 186gr/day compared to 32 to 82 gr/day of red meat and 34 to 82 gr/day of processed meat. In the studies using 24HR recall, the percent of participants reporting consuming poultry was highest and Q4 intake of poultry was 146 and 172 gr/day in Mabat Zahav and the NNS studies, respectively.

**Table 3:** Meat consumption (median for quartile) by study

		**Total Meat**				**Total Red Meat**					**Total Processed Meat**					**Total Poultry**				
		(gr/day), median for each quartile																		
**Study**	**Consumers**	**Q1**	**Q2**	**Q3**	**Q4**	**Consumers**	**Q1**	**Q2**	**Q3**	**Q4**	**Consumers**	**Q1**	**Q2**	**Q3**	**Q4**	**Consumers**	**Q1**	**Q2**	**Q3**	**Q4**
**Studies using FFQ**																				
NICCCS *n* = 13,357	98%	43.5	76.5	106.0	155.5	84%	0	17.5	27.9	67.8	93%	8.8	27.6	51.8	81.9	96%	17.5	51.7	62.5	98.8
IIHD *n* = 1058	99%	43.3	68.3	93.9	134.9	96%	10.7	26.5	40.9	67.9	81%	0	8.2	16.5	34.3	93%	11.2	30.0	49.6	90.3
Ovary *n* = 1763	97%	28.1	56.9	82.1	117.0	73%	0	3.6	13.3	32.1	92%	3.6	15.7	30.0	55.7	96%	17.9	41.6	61.4	94.3
GOH *n* = 632	100%	72.8	123.9	167.9	246.1	90%	1.4	17.9	35.7	78.9	91%	3.6	17.9	33.2	64.3	99%	42.9	77.1	115.3	185.7
HDS *n* = 1098	99%	54.1	91.7	121.4	165.9	92%	2.5	15.9	30.0	55.5	92%	3.6	21.1	37.9	72.9	97%	31.8	65.7	89.3	131.1
**Studies using 24HR recall**																				
NNS *n* = 986	78%	0	51.8	101.0	190.5	28%	0	0	16	82	16%	0	0	0	54	59%	0	37.9	80	171.5
Mabat- Zahav *n* = 1751	72%	0	48	93	191	27%	0	0	16	79	15%	0	0	0	37	49%	0	0	58	146

*Abbreviations*: *FFQ* Food Frequency Questionnaire, *IIHD* Israel Ischemic Heart Study, *GOH* Glucose Intolerance, Obesity, and Hypertension study, *NICCCS* Northern Israel Cancer Case Control studies, *HDS* Hadera District Study, *NNS* the Negev Nutritional Study

Table 4 presents intakes of meat categories (gr/day) by nutritional assessment type and sex. In general, men reported consuming more meat than women did. This corroborated with a greater mean total energy intake found in men then in women, for both types of nutritional assessment. In studies using FFQ, women reported consuming more processed poultry and organ meats than men.

**Table 4:** Intake (gram per day) for each meat category according to questionnaire type and sex

Weight (gram per day]	FFQ Weighted Mean^a^ ± SE^b^			24-HR recall Weighted Mean ^a^ ± SE^b^		
Food Category	Total	Men	Women	Total	Men	Women
n	26,909	13,987	12,922	2,733	1,268	1,465
Total energy intake (Kcal/day)	1,940 ± 3.9	2,412 ± 6.5	1,669 ± 4.8	1,509 ± 12.1	1,718 ± 19.0	1,329 ± 13.8
Total Meat	94.6 ± 0.4	108.8 ± 0.8	89.3 ± 0.4	92.0 ± 1.9	110.0 ± 3.3	77.0 ± 2.1
Total processed meat	37.0 ± 0.2	31.5 ± 0.4	39.1 ± 0.3	10.0 ± 0.7	13.0 ± 1.1	8.0 ± 0.8
Total Red meat ^c^	27.0 ± 0.2	38.6 ± 0.5	22.9 ± 0.2	25.0 ± 1.1	33.0 ± 1.9	18.0 ± 1.1
Unprocessed Red Meat (beef/pork/lamb)	10.4 ± 0.1	17.3 ± 0.3	8.5 ± 0.1	14.0 ± 0.9	18.0 ± 1.6	11.0 ± 1.0
Processed beef/pork	8.1 ± 0.1	10.3 ± 0.2	9.4 ± 0.1	1.7 ± 0.3	2.8 ± 0.6	0.7 ± 0.2
Ultra-processed beef/pork	3.1 ± 0.07	3.9 ± 0.13	1.2 ± 0.05	2.7 ± 0.3	3.7 ± 0.4	1.6 ± 0.3
Total Poultry ^d^	58.0 ± 0.3	67.0 ± 0.5	54.0 ± 0.3	66.0 ± 1.9	77.0 ± 3.2	57.0 ± 2.1
Unprocessed Poultry (Chicken/Turkey/Duck)	35.0 ± 0.2	50.0 ± 0.4	29.0 ± 0.3	51.0 ± 2.2	53.0 ± 2.4	45.0 ± 2.5
Processed poultry	22.0 ± 0.2	16.0 ± 0.3	23.0 ± 0.2	2.5 ± 0.4	2.5 ± 0.6	2.3 ± 0.5
Ultra-processed poultry	2.1 ± 0.04	3.6 ± 0.13	1.6 ± 0.04	3.1 ± 0.3	3.1 ± 0.4	3.0 ± 0.4
Fish	35.0 ± 0.5	44.7 ± 0.9	31.0 ± 0.6	22.0 ± 1.2	25.0 ± 2.0	20.0 ± 1.4
Organ meats	5.0 ± 0.07	3.2 ± 0.09	8.5 ± 0.10	2.5 ± 0.3	2.9 ± 0.5	2.0 ± 0.4

^a^The weighted mean was calculated by weighting the mean of each separate study according to its standard error, the weight being$\frac{1}{{se}^{2}}$

^b^The standard error was calculated as 1 divided by the square root of the sum of weights

^c^Beef/pork/lamb

^d^Chicken/Turkey/Duck

The BMI and nutrient intake by quartile of total meat consumption are shown in Table 5. In general, absolute nutrients consumption were greater with greater intake of total meat, indicating a generally higher food intake. Percent energy from carbohydrates, and intakes per 1000kcal of dietary fiber, iron, calcium and folic acid were negatively associated with increasing quartiles of meat intake (p _linear trend_ < 0.0001 for all) while median BMI was greater with greater meat intake (from 26.7 to 27.2, p _linear trend_ < 0.0001).

**Table 5:** BMI, energy intake, and selected nutrients intake by quartiles of total meat consumption in pooled FFQ data

Characteristic		Total Meat Consumption**			
		Q1	Q2	Q3	Q4
	n^a^	Mean (se)^b^	Mean (se)^b^	Mean (se)^b^	Mean (se)^b^
BMI	26,308	26.7 (0.08)	27.2 (0.08)	27.2 (0.08)	27.2 (0.08)
Energy (kcal/day)	25,085	1,486 (7.5)	1,623 (7.2)	1,773 (7.9)	2,072 (9.1)
Protein (gr/day)	25,087	72 (0.4)	83 (0.3)	92 (0.4)	109 (0.4)
Protein density (% of total energy)	25,082	18.5 (0.06)	19.5 (0.05)	19.7 (0.06)	20.7 (0.06)
Carbohydrates (gr/day)	25,087	191 (1.1)	199 (1.1)	213 (1.2)	237 (1.3)
Carb density (% of total energy)	25,082	51.2 (0.1)	49.0 (0.1)	47.9 (0.1)	45.6 (0.1)
Dietary fibers (gr/day)	15,156	13 (0.1)	13 (0.1)	14 (0.1)	15 (0.1)
Dietary Fibers density (gr/1000 kcal)	15,156	9.0 (0.06)	8.4 (0.05)	8.0 (0.05)	7.4 (0.04)
Fat (gr/day)	25,087	50 (0.3)	56 (0.3)	62 (0.3)	77 (0.4)
Fat density (% of total energy)	25,082	30.2 (0.09)	31.2 (0.09)	31.9 (0.08)	33.7 (0.09)
Saturated fat (gr/day)	25,087	11.2 (0.1)	13.1 (0.1)	15.2 (0.1)	19.7 (0.1)
Saturated fat density (% total energy)	25,082	7.2 (0.04)	7.7 (0.04)	8.1 (0.04)	8.9 (0.04)
Cholesterol (mg/day)	22,197	183 (1.6)	226 (1.4)	265 (1.5)	348 (2.3)
Alcohol (gr/day)	16,170	0.9 (0.05)	1.1 (0.06)	1.3 (0.07)	1.8 (0.09)
Calcium (mg/day)	12,761	802 (6.2)	806 (5.8)	829 (6.1)	909 (6.9)
Calcium density (mg/1000 kcal)	12,761	556 (3.1)	499 (2.7)	461 (2.6)	425 (2.5)
Iron (mg/day)	14,524	9.5 (0.06)	10.3 (0.06)	11.2 (0.06)	13.1 (0.07)
Iron density (mg/1000 kcal)	14,524	6.6 (0.03)	6.5 (0.03)	6.4 (0.02)	6.4 (0.02)
Folic acid (mcg/day)	13,426	218 (1.8)	230 (1.6)	242 (1.7)	270 (1.9)
Folic acid density (mcg/1000 kcal)	13,426	152 (0.9)	147 (0.8)	142 (0.8)	135 (0.8)

^**^p-value of a test for linear trend (see appendix #2) across the quartiles < 0.001 for all characteristics

^a^differences in number of participants with available data are due to missing information on specific nutrients in some of the studies

^b^For each study separately, the mean and standard error of intake were calculated in each quartile of meat consumption. Then, for each quartile, a weighted mean and standard error were calculated as in Table 4

## Discussion

We report here on the dietary intake of more than 20,000 participants of seven studies, using different nutritional questionnaires and food composition tables and spanning over five decades. This methodological heterogeneity presented a great challenge to the nutritional data harmonization, especially since the main exposure of interest was on the food level (red and processed meat). Several strategies were employed to deal with this challenge, including revision of food level data, standardizing of dietary and sociodemographic variables and the creation of a uniform system of food grouping that was applied to all studies.

### Nutritional data harmonization

This methodological paper is part of a larger project aimed at studying the association of red and processed meat with gastrointestinal cancers and for this reason was focused on meat intake. We found that over 95% of participants reported consuming any meat in studies using FFQ, and that consumption of poultry was the highest of all meat sub-categories in all studies. Total meat intake was similar across studies using FFQ and 24HR recall questionnaires, while in the sub-categories of meat more differences were observed. In the processed meat category higher intakes were found in studies using FFQ, probably due to the different classification of specific meat items owing to the lack of full information on mode and place of preparation in the FFQ.

Other studies applying harmonization to previously collected data faced similar challenges and similarly aggregated food items into food groups to create a uniform system [22, 23]. Most previous studies merged dietary data of FFQs only [22–24], Olsen et al. harmonized data from two large birth cohorts that used similar questions and software for calculation of food intake, demonstrating the advantage of pooling data from studies with comparable nutritional questionnaires. Similarly to the current study, the EURALIM collaboration attempted to harmonize dietary data of six surveys, of them one used a 24HR recall, one used repeated 1-day diet records and the remaining four used FFQs, the authors concluded that the methodological heterogeneity was too great to directly compare dietary measures [25]. Following the harmonization of data in the current study, we observed similarities between the two types of nutritional questionnaires in mean intakes of total meat, red meat and poultry, while differences were apparent in sub-groups (i.e., processed meat). These differences were considered and lead to a separate analysis according to nutritional tool type.

### Dietary intake

In the current study, red meat intake was 22.9gr/day among women and 38.6 gr/day among men in studies using FFQ, while in studies using 24HR recall it was 18.0 and 33.0gr/day in women and men respectively. This higher meat consumption in men aligns with the higher total energy intake in men than in women seen both in our study and as reported previously [26]. As for the slightly higher consumption of processed meat, mainly due to processed poultry consumption and organ meats, of women vs. men, according to the FFQ, this may be explained by reporting differences. A systematic review by Lee (2016) highlight the impact of gender differences in FFQ, with greater inaccuracy in dietary intake assessment in women [27]. In addition, pork was consumed by very few (data not shown) and alcohol intake was low. Thus, this study confirms findings from previous reports and studies on the dietary habits of the Israeli population, which include avoidance of pork meat and of alcohol. These habits are related mainly to religious observance laws of the Jewish and Muslim population, and reflect the older age groups of participants in the studies. According to the OECD's meat consumption indicator, comparing food purchasing per capita, Israel is leading in purchases of poultry, while pork purchases are among the lowest worldwide [28]. While in Israel pork consumption reached only 1.2 kg/capita in 2019, in USA the figure was 23.8 kg/capita. For poultry, Israelis consume 68.7 kg/capita compared to 50.9 kg/capita in USA. Beef annual consumption was close between Israel and USA, with 24.1 and 26.0 kg/capita, respectively. In studies of individual dietary data, average red meat intake in the U.S., reported by the NHANES III, was 69.8gr daily [29]. In European countries intakes ranged from 71 gr/day in the UK, up to 97.6 gr/day in Sweden [30, 31]. A recent Israeli case–control study reported red meat intake of approximately 23 gr daily (1.3 portions/week) by Jewish participants and 53 gr daily (3 portions/week) by Arab participants [32]. The relatively low meat intake and other unique dietary habits highlight the need to explore the relationship between dietary intake and health outcomes in ethnically and culturally diverse populations.

We further compared nutrient intake of participants by quartiles of total meat consumption among studies using FFQ; we found that in absolute terms those who consumed more meat ate more of all nutrients. When examining nutrient intake in relative terms (as percent of total energy or per 1000kcal) we found that those who consumed more meat ate relatively more protein and fat and less fiber, calcium, iron and folic acid. Our finding of a relatively lower consumption of iron among those in the higher quartiles of meat intake is surprising as meat is a key source of this nutrient. However, in our population, poultry, which is relatively low in iron [33], was the main type of meat consumed. These differences in nutrient intake indicate that consuming more meat may be linked to other food choices and demonstrate how the general dietary pattern may differ between low and high meat consumers.

An important advantage of the current collaboration is the high number of participants, with diverse sociodemographic backgrounds that allowed us to explore a wide range of diets. The use of previously collected data enabled us to efficiently study long term effects of the diet, in a relatively short time and low costs. In addition, inclusion of 7 studies, each conducted in a different decade, allowed representation of the changing dietary habits and the composition of foods, which occurred over a long period of time.

The current study has several limitations, mainly the heterogeneity of the nutritional questionnaires used by the different studies (q-FFQ and 24HR recall) that may lead to discrepancies in exposure assessment. In Israel, meat is not consumed daily by most people, poultry being more commonly consumed (as seen in Table 3, %consumers). The 24HR instrument fails to capture intake of episodically consumed foods (i.e., those that are not consumed every day by most people), while the FFQ has the strength of querying about long-term intake, thereby aiming to obtain data on usual intake with a single administration. This explains the differences in the proportion of consumers of meat between the two nutritional instruments. Nevertheless, an analysis performed according to questionnaire type revealed similarities in patterns of food consumption by sex in the two types of questionnaires used in our study (as seen in Table 4). The collection of nutritional data only once throughout the participant's life, which provides a snapshot of the person's dietary intake [34] is yet another limitation of the present study.

## Conclusions

The methodology described in this paper may be applicable in other settings and we demonstrate the feasibility of addressing challenges in harmonization of nutritional databases. Using food level dietary exposure data from different studies, offers a unique opportunity to examine links between a wide range of dietary exposures and the outcome of interest. Studying populations with a unique food culture is important, as it allows looking into otherwise rare exposures, and as it enables tailoring of dietary recommendations that are best suited to the population of interest.

### Supplementary Information

Supplementary Material 1.

Supplementary Material 2.

## Data Availability

Data will be available upon reasonable request.
