# Supplementary material for: Methodology and challenges for harmonization of nutritional data from seven historical studies
Source: Nutr J. 2024 Aug 6;23:88. doi: 10.1186/s12937-024-00976-8 (PMC11302319; doi:10.1186/s12937-024-00976-8)
Supplement: Supplementary file 1 — Supplementary Material 1. [file 12937_2024_976_MOESM1_ESM.docx]

Appendix Table 1: Additional food groups available for 5,598 individuals, according to sex and nutritional assessment type

| Studies using 24-h recall  Mean±SD  Median [q1, q3] | | | Studies using FFQ  Mean±SD  Median [q1, q3] | | | Studies using FFQs Weight (gram/day] |
| --- | --- | --- | --- | --- | --- | --- |
| Women (n) | Men (n) | Total (n) | Women (n) | Men (n) | Total (n) | Food Category, (gram/day) |
| 1467 | 1270 | 2737 | 2312 | 549 | 2861 |  |
| 153±116  126 [73, 210] | 190±135  160 [92, 256] | 170±126  144 [81, 230] | 177±108  150 [104, 222] | 336±175  302 [212, 429] | 207±139  168 [112, 270] | Bread and cereals |
| 21±115  0 [0, 40] | 27±45  0 [0, 56] | 24±89  0 [0, 50] | 15±17  11 [5, 21] | 27±27  19 [8, 39] | 18±20  14 [7, 23] | Eggs and egg products |
| 227±176  200 [83, 325] | 226±190  200 [75, 330] | 226±183  200 [80, 330] | 294±193  260 [162, 387] | 271±195  238 [136, 342] | 290±193  257 [157, 377] | Milk and milk products |
| 228±223  175 [93, 306] | 254±282  195 [92, 342] | 240±252  186 [92, 319] | 259±163  220 [148, 325] | 287±194  236 [159, 362] | 264±170  223 [150, 334] | Fruits |
| 247±222  194 [91, 348] | 290±275  230 [105, 397] | 267±249  211 [100, 369] | 293±176  259 [169, 376] | 330±180  302 [205, 417] | 300±178  265 [177, 385] | Vegetables |
| 30±80  0 [0, 12] | 46±95  0 [0, 50] | 38±88  0 [0, 30] | 14±22  7 [1, 18] | 21±26  14 [6, 26] | 16±23  9 [2, 20] | Legumes |

Appendix #2

Linear trend across the quartiles was calculated as follow:

Let x1, x2, x3, x4 be the weighted mean in quartiles 1-4; let their se’s be s1, s2, s3, s4.

The change in weighted mean per quartile was calculated as: (3*x4 + x3 – x2 – 3*x1)/2 = B.

The standard error of B was calculated as: √[(9*s4^2 + s3^2 + s2^2 +9*s1^2)/4] = SE(B).

We then calculated z = B/SE(B) and compared to normal distribution to get a p-value.

Appendix #3

See attached
